# Cognition and Social Behaviors Related to COVID-19 Among Students in Medical Colleges: A Cross-Sectional Study in Guangdong Province of China

**DOI:** 10.3389/fpubh.2022.782108

**Published:** 2022-03-18

**Authors:** Qiu Zhang, Xiaoya Lu, Mengxin Liao, Xinyue Zhang, Liqing Yao

**Affiliations:** ^1^School of Medical Business, Guangdong Pharmaceutical University, Guangzhou, China; ^2^Research Base of Regulatory Science for Medical Products, Guangzhou, China; ^3^School of Pharmacy, Guangdong Pharmaceutical University, Guangzhou, China

**Keywords:** COVID-19 pandemic, cognition, social behavior, medical college students, China

## Abstract

**Background:**

The COVID-19 pandemic is a public health emergency of international concern. This study aimed to describe the cognition and social behaviors related to COVID-19 among medical college students in China and to explore the relevant factors that have affected individual social behaviors. The study could enrich practical research on the social behaviors of college students during the COVID-19 pandemic.

**Methods:**

From February to April 2020, online questionnaire survey was conducted meticulously. Based on their majors, the students were divided into a medical student group (249 cases) and a near-peer medical student group (397 cases). Descriptive statistics was used to elaborate the cognition related to the pandemic and the status quo of social behaviors among these students. A multiple linear regression model was established to analyze the relevant factors affecting individual social behaviors from various perspectives during the pandemic.

**Results:**

Regarding the cognition situation: 76.32% of those surveyed had good pandemic awareness, and the average general cognition score was 30.55 ± 3.17 points. In terms of social behaviors, the average scores for purposive rational actions and affective actions during the outbreak were relatively high, scoring 8.85 ± 1.72 points (>10 points) and 4.32 ± 1.41 points (>6 points), respectively, while the average value rational actions score was relatively low at 5.95 ± 1.90 points (>10 points). The results of the multiple linear regression model showed that urban college students had higher scores for purposive rational actions; college students with the CCP membership had higher value rational actions scores; school and major were also significant factors affecting affective actions scoring. The COVID-19 cognition score had a significant effect on the social behavior score in all dimensions (*P* < 0.001).

**Conclusions:**

The cognition of COVID-19 among students in Chinese medical colleges was good, and pandemic cognition was an important factor that affected individual social behaviors. Universities and colleges should strengthen the publicity and education of knowledge related to COVID-19, guide students to internalize their knowledge of the pandemic into positive behaviors, and help to win the battle of pandemic prevention and control.

## Introduction

On January 30, 2020, the World Health Organization (WHO) announced that the pandemic would be listed as a “Public Health Emergency of International Concern (PHEIC)”. According to WHO data, as of 0:00 on March 16, 2020, Central European time (7:00 March 16, Beijing time), there were 164,837 confirmed cases of COVID-19 worldwide, including 81,077 in China and 83,760 outside of China. The global “fight against COVID-19” had begun. COVID-19 had attracted the attention of people all over the world since then.

This major public health emergency is not only a challenge to modern medicine and social health service systems but also a solid test of risk perception and health literacy among professionals. Studies have shown that individual cognition and social behaviors are closely related to the effectiveness of infectious disease prevention and control (1). As an important reserve on the frontline of the national anti-pandemic struggle, social behaviors of medical college students significantly affect the prevention and control of the pandemic. However, information and professional knowledge cannot always be internalized into individual behaviors that can actively respond to the pandemic. The present paper tried to clarify these three issues: (1) How to evaluate the cognition and describe the social behaviors related to COVID-19 among medical college students in China? (2) Does the COVID-19 cognition level of medical college students in China influence their social behaviors? (3) What are the factors that influence social behaviors in different dimensions among medical college students in China?

Nonetheless, social studies on COVID-19 have been relatively limited, as the main focus of research has been medical, such as clinical research (2–4), epidemiology (5–7), and vaccines (8–10). In the post-pandemic era, the importance of a country's pandemic control performance and strategic planning experience has become more and more prominent, and the gap in the construction of medical and health services has also been exposed to the public. It is particularly vital to conduct research on COVID-19 from a sociological perspective. In previous studies on major public health emergencies, Cha et al. investigated the cognition and behaviors of medical students in relation to SARS during the 2003 SARS epidemic. It was argued that medical students generally had a high degree of mastery of clinical and etiological knowledge, transmission routes, and prevention knowledge related to the SARS epidemic, as well as better pandemic prevention and control behaviors (11). Pan et al. studied the knowledge and behaviors of bird flu prevention and control of medical college students and found that the most of medical students had satisfactory knowledge of bird flu. In a survey of related questions, the overall average score was 11.08 ± 3.21 points (>22 points). It was found that the students paid more attention to self-protection behaviors and also volunteered to participate in the prevention and control of bird flu (12). In a study of the COVID-19 pandemic, Alsoghair et al. found that Saudi Arabian medical students exhibited a high level of COVID-19 knowledge (83.9%) and an overwhelmingly high degree of preventative behaviors to protect them against COVID-19 (94.1%). Additionally, a significant positive correlation between pandemic knowledge and self-reported preventive behavior scores was reported (13). Taghrir et al. came to a similar conclusion: Iranian medical students had high awareness of COVID-19 knowledge and prevention and control behaviors (14). However, few research have been conducted on cognition and social behaviors related to COVID-19. In-depth exploration of the relationship between them in the Chinese medical student group is also rare. In view of this, we selected Guangdong province, which has the second highest cumulative number of confirmed cases during the COVID-19 epidemic and was also the hardest hit place in the 2003 SARS epidemic, to conduct our survey, aiming to explore the relevant factors affecting individual social behaviors during the pandemic.

As the most populous country of the world, China has made great achievements in the prevention and control of the pandemic, in which medical students have made solid contributions. Medical students are also a key factor in the grassroot public health prevention and control work after the pandemic, as well as the reservoir of future health force. Therefore, it is of great significance to investigate and analyze the awareness, attention, and response as well as the cooperation and empathy capability of Chinese medical college students during the pandemic. This study is expected to make the following contributions: (1) Make more people aware of the COVID-19 cognition of students in Chinese medical colleges. (2) Explore the factors influencing the social behaviors of medical college students during the COVID-19 pandemic.

## Materials and Methods

### Objectives

An online survey was conducted among six medical colleges in Guangdong Province, China: Sun Yat-sen University School of Medicine, Guangzhou University of Chinese Medicine, Guangzhou Medical University, Guangdong Medical University, Southern Medical University, and Guangdong Pharmaceutical University. With reference to the national discipline classification and the research of some domestic scholars, the participants were divided into two groups: medical students (majors with a bachelor degree in medicine after graduation, including clinical medicine, preventive medicine, nursing and other majors) and near-peer medical students (majors other than medicine in medical college, such as English language, law, health management, and pharmacy, etc.) (15). Data were collected online from 1 February 2020 to 28 April 2020. Convenience sampling was used to collect maximized samples. Informed consent and responses to the questionnaire were obtained from 646 of the 662 (97.6%) students who were surveyed.

### Methods

#### Measures

We developed an online, self-administrated questionnaire that focused on (1) general demographic characteristics; (2) COVID-19 related knowledge; and (3) social behaviors during the pandemic. The design of the questions was based on the “novel coronavirus infection pneumonia treatment program (Trial Fifth Edition)” released by the National Health Committee and the “New Coronavirus Transmission Route and Prevention Guidelines” issued by the Centers for Disease Control (CDC) in China, which includes four dimensions: clinical features (possible symptoms), epidemiological characteristics (possible routes of transmission, source of infection, development status of specific drugs and vaccines, precaution, types of susceptible population, types of prevention and control measures), etiological characteristics (comparison with “SARS,” the substance or method of inactivating the virus), and diagnostic criteria (meeting the conditions of a suspected case). We set 36 scores for this category in total and summed the scores obtained. If the scores were more than 28.8 (80%), a good level of cognition was indicated, so poor cognition was indicated for scores were below 28.8. The design of the questions was based on “Basic Concepts of Sociology,” written by the famous German sociologist Max Weber (1864–1920) (16) to classify social behaviors. These behaviors are divided into three dimensions: (1) purposive rational actions (the actors are guided by ends, means and incidental consequences, and after rational weighing, they choose their own goals from a series of goals); (2) value rational actions (the actors ignore the foreseeable consequences, but only to fulfill their instructions on duty, honor, beauty and religious pronouncement); (3) affective actions (the social behavior determined by current emotions or feelings). The maximum score for each dimension was 10, 10, and 6, respectively.

#### Quality Control

During the questionnaire design phase, we adopted the Delphi method to screen factors repeatedly, and demonstrated the accuracy of the content and logic of the questionnaire. 10 Delphi panelists were experts in management, preventive medicine and sociology. They were represented by the disciplines of management, clinical medicine, public health, and sociology. In each questionnaire module, experts must express their opinion by responding with Disagree/Neutral/Agree. An iterative Delphi round will take place before a final unanimous consensus result was reached. And the collective opinions of each round will be fed back to the members as references for the next round. No experts quitted during the whole process, and the Expert positivity coefficient of each round was 100% (see Table 1). The preliminary questionnaire was distributed to a small range of respondents in the form of a presurvey, aiming to revise and finalize the content of the questionnaire.

**Table 1:** Consensus-based views and opinions of the Delphi panelists on the questionnaire sections.

**Items**	**Round 1**			**Round 2**			**Round 3 (finalized)**		
	**Disagree**	**Neutral**	**Agree**	**Disagree**	**Neutral**	**Agree**	**Disagree**	**Neutral**	**Agree**
COVID-19 cognition	0 (0.0)	3 (30.0)	7 (70.0)	0 (0.0)	0 (0.0)	10 (100.0)	0 (0.0)	0 (0.0)	10 (100.0)
Purposive rational actions	0 (0.0)	2 (20.0)	8 (80.0)	0 (0.0)	0 (0.0)	10 (100.0)	0 (0.0)	0 (0.0)	10 (100.0)
Value rational actions	0 (0.0)	3 (30.0)	7 (70.0)	0 (0.0)	1 (10.0)	9 (90.0)	0 (0.0)	0 (0.0)	10 (100.0)
Affective actions	1 (10.0)	4 (40.0)	5 (50.0)	0 (0.0)	2 (20.0)	8 (80.0)	0 (0.0)	0 (0.0)	10 (100.0)
Basic information	0 (0.0)	4 (40.0)	6 (60.0)	0 (0.0)	0 (0.0)	10 (100.0)	0 (0.0)	0 (0.0)	10 (100.0)

### Statistical Analysis

Data was analyzed by Excel and the SPSS statistical software (version 22.0; SPSS Inc., Chicago, IL, USA). The Mann–Whitney U test was employed to compare groups in a univariate analysis, and the independent sample non-parametric Kruskal–Wallis H test was used for multiple sets of data with uneven variance. In the multi-factor analysis, the social behavior score was used as the dependent variable to establish a multiple linear regression model, where the independent variables were general demographic characteristics and cognition variables. Spearman's correlation test was applied to analyze the correlations among purposive rational actions, value rational actions, and affective actions scores. All statistical tests were two-tailed tests, and the significance level was set at α = 0.05.

## Results

### Basic Information

Of the 646 participants, 205 were male and 441 were female. 249 of them were medical students and 397 were near-peer medical students. Non-Only-Child students accounted for the majority (75.23%) of respondents, and 390 respondents were born in towns. The grade distribution was as follows: freshman 18.42, sophomore 19.97, junior 50.77, senior 6.97, senior fifth 0.15, and students with a master's degree or above 3.72%. In terms of political orientation, there was a high proportion of communist youth league members (72.29%). The proportion of students whose parents engaged in occupations as doctors or nurses was 7.12%. 77.55% of the respondents lived in areas with no confirmed cases. The average score of cognition related to COVID-19 was 30.55 ± 3.17, and 76.32% had a high level of cognition. The average score of purposive rational actions was 8.85 ± 1.72, the value rational actions was 5.95 ± 1.90, and the affective actions was 4.32 ± 1.41.

### COVID-19 Cognition

The correct answer rate of research subjects regarding COVID-19 knowledge was between 60 and 80%. However, regarding to the clinical characteristics of COVID-19 and SARS, the correct answer rates were relatively lower at 28.33 and 39.01%, respectively. The accuracy for questions on the substance or method of inactivating the virus was the lowest, only 4.33%. As for the possible symptoms of COVID-19, 34.94% of medical students answered correctly, while 24.18% of near-peer medical students answered correctly, and the difference was statistically significant (χ^2^ = 8.723, *P* < 0.05). 57.18% of near-peer medical students were aware of the possible transmission route of COVID-19, while 46.99% of medical students were aware of it, and the difference was statistically significant (χ^2^ = 6.384, *P* < 0.05) too. Regarding the prevention and control measures of COVID-19, medical students showed a higher cognition accuracy rate (72.29%), while near-peer medical students had an average score of 60.96%. The difference was also statistically significant (χ^2^ = 8.311, *P* < 0.05) (see Table 2).

**Table 2:** Accuracy of answers to questions on COVID-19-related knowledge.

**Knowledge**		**Total (%)**	**Medical students (%)**	**Near-peer medical students (%)**	***χ^2^***	***P***
Clinical features	1) Possible symptoms	183 (28.33)	87 (34.94)	96 (24.18)	8.723	0.003^**^
Epidemiological characteristics	2) Possible routes of transmission	344 (53.25)	117 (46.99)	227 (57.18)	6.384	0.012^*^
	3) Source of infection	417 (64.55)	151 (60.64)	266 (67.00)	2.705	0.100
	4) Development status of specific drugs and vaccines	492 (76.16)	194 (79.91)	298 (75.06)	0.684	0.408
	5) Precaution	465 (71.98)	171 (68.67)	294 (74.06)	2.197	0.138
	6) Types of susceptible population	443 (68.58)	163 (65.46)	280 (70.53)	1.823	0.177
	7) Types of prevention and control measures	423 (65.48)	180 (72.29)	242 (60.96)	8.311	0.004^**^
Etiological characteristics	8) Comparison with “SARS”	252 (39.01)	96 (38.55)	156 (39.29)	0.035	0.851
	9) The substance or method of inactivating the virus	28 (4.33)	7 (2.81)	21 (5.29)	2.267	0.132
Diagnostic criteria	10) Meet the conditions of a suspected case	486 (75.23)	188 (75.50)	298 (75.06)	0.016	0.900

** *P < 0.01*,

* *P < 0.05*.

### Social Behaviors of Students From Chinese Medical Colleges During the COVID-19 Pandemic

#### The Status of Purposive Rational Actions

The subjects showed acceptable levels of purposive rational actions related to the COVID-19 pandemic and corresponding preventive measures to maintain their own health. More than 80% of the behaviors, such as protective behaviors out-of-home, hygiene habits, and living habits, were adopted. Protective behaviors at home displayed the lowest adoption proportion at 59.60, being carried out by 53.01 of medical students, and 63.73% of near-peer medical students, and the difference was statistically significant (χ^2^ = 7.298, *P* < 0.05) (see Table 3).

**Table 3:** Basic status of three social behaviors during the COVID-19 pandemic.

**Dimensions**	**Topics**	**Total (%)**	**Medical students (%)**	**Near-peer medical students (%)**	***χ^2^***	***P***
Purposive rational actions	hygiene habits	570 (88.24)	218 (87.55)	352 (88.66)	0.183	0.669
	living habits	518 (80.19)	201 (80.72)	317 (79.85)	0.074	0.786
	protective behaviors at home	385 (59.60)	132 (53.01)	253 (63.73)	7.298	0.007^*^
	protective behaviors out-of-home.	594 (91.95)	226 (90.76)	368 (92.70)	0.772	0.380
Value rational actions	voluntary activity participation	51 (7.89)	21 (8.43)	30 (7.56)	0.162	0.687
	information learning and dissemination	201 (31.11)	84 (33.73)	117 (29.47)	1.298	0.255
	pandemic decision-making	251 (38.85)	94 (37.75)	157 (39.55)	0.208	0.649
Affective actions	medical work approval	442 (68.42)	172 (69.08)	270 (68.01)	0.081	0.777
	career planning	199 (30.80)	117 (46.99)	82 (20.65)	49.781	<0.001^**^
	no feelings	7 (1.08)	1 (0.40)	6 (1.51)	1.758	0.185

** *P < 0.01*,

* *P < 0.05*.

#### The Status of Value Rational Actions

As for value rational actions, our subjects showed room for improvement. It was reported that most of them had not actively participated in the anti-pandemic movement. The proportions of respondents who had implemented the value rational actions such as voluntary activity participation, information learning and dissemination, and pandemic decision-making were 7.89, 31.11, and 38.85%, respectively (see Table 3).

#### The Status of Affective Actions

The emotions generated by the subjects during the pandemic became more positive through the visualization of behavioral decision-making. These behaviors could be classified into two categories: the high degree of approval for medical work (68.42%) and the career planning in the medical field (30.80%). The proportion of career planning implemented by medical students (46.99%) was significantly higher than that in the near-peer ones (20.65%). And the difference was statistically significant (χ^2^ = 49.781, *P* < 0.001). There was no statistical significance between medical students and near-peer ones for the non-positive emotion of “no feelings” (see Table 3).

### Comparison of Social Behavior Scores of Different Groups Among Medical College Students in China

Three different dimensions of social behavior scores were used as dependent variables, and 11 factors were used as independent variables, which included not only the pandemic cognition grouping variables and the cognitive scoring numerical variables, but also the variables of gender, major, and political orientation (see Table 4).

**Table 4:** Comparison of social behavior scores of different groups of medical college students in China.

**Variables**	***N***	**Purposive rational actions**			**Value rational actions**			**Affective actions**		
		**Score**	**Statistic**	***P***	**Score**	**Statistic**	***P***	**Score**	**Statistic**	***P***
**Gender**			−0.969	0.332^a^		−1.537	0.124^a^		−0.641	0.522^a^
Male	205	8.78 ± 2.06			5.74 ± 2.09			4.31 ± 1.58		
Female	441	8.89 ± 1.54			6.05 ± 1.80			4.32 ± 1.32		
**Only child**			−0.288	0.774^a^		−0.321	0.748^a^		−0.946	0.344^a^
Yes	160	8.94 ± 1.63			5.91 ± 1.94			4.22 ± 1.44
No	486	8.82 ± 1.75			5.96 ± 1.89			4.35 ± 1.39
**School**			3.805	0.578^b^		4.408	0.492^b^		28.631	<0.001^b^^**^
Sun Yat-sen University School of Medicine	15	8.13 ± 2.33			5.40 ± 1.72			3.53 ± 1.46		
Guangzhou University of Chinese Medicine	98	8.90 ± 1.60			5.82 ± 1.74			4.69 ± 1.26		
Guangzhou Medical University	30	9.03 ± 1.43			6.20 ± 2.30			4.93 ± 1.44		
Guangdong Medical University	39	8.74 ± 1.85			5.59 ± 2.09			4.21 ± 1.58		
Southern Medical University	45	8.98 ± 1.16			6.24 ± 2.01			4.71 ± 1.49		
Guangdong pharmaceutical University	419	8.85 ± 1.79			5.98 ± 1.88			4.19 ± 1.38		
**Major**			−2.046	0.041^a^^*^		−0.235	0.814^a^		−6.059	<0.001^a^^**^
Medical student	249	8.76 ± 1.72			5.94 ± 2.02			4.71 ± 1.43		
Near-peer medical student	397	8.91 ± 1.72			5.95 ± 1.82			4.08 ± 1.34		
**Grade**			10.620	0.059^b^		8.516	0.130^b^		2.856	0.722^b^
Freshman	119	8.74 ± 1.91			5.71 ± 1.87			4.20 ± 1.44		
Sophomore	129	8.84 ± 1.52			5.77 ± 1.78			4.33 ± 1.36		
Junior	328	8.79 ± 1.80			6.03 ± 1.94			4.32 ± 1.44		
Senior	45	9.20 ± 1.49			6.56 ± 2.00			4.64 ± 1.21		
Senior fifth	1									
Master's degree and above	24	9.58 ± 0.93			5.71 ± 1.60			4.25 ± 1.36		
**Birthplace**			−2.026	0.043^a^^*^		−1.470	0.141^a^		−0.940	0.347^a^
Town	390	8.96 ± 1.64			6.03 ± 1.95			4.37 ± 1.38		
Rural area	256	8.68 ± 1.83			5.82 ± 1.80			4.24 ± 1.44		
**Political orientation**			3.731	0.292^b^		21.926	<0.001^b^^**^		10.316	0.016^b^^*^
Masses	43	9.12 ± 1.60			5.79 ± 2.11			4.63 ± 1.38		
Communist youth league member	467	8.76 ± 1.85			5.77 ± 1.87			4.21 ± 1.43		
Active applicants of party membership	87	9.14 ± 1.30			6.39 ± 1.79			4.69 ± 1.32		
Probationary party member/party member	49	9.00 ± 1.12			7.00 ± 1.66			4.41 ± 1.22		
**Whose parents engaged in occupations as doctors or nurses**			−0.413	0.679^a^		−1.639	0.101^a^		−1.830	0.067^a^
Yes	46	8.96 ± 1.55			6.41 ± 1.92			4.72 ± 1.17		
No	600	8.84 ± 1.74			5.91 ± 1.89			4.29 ± 1.42		
**Surrounding confirmed cases**			3.841	0.147^b^		2.767	0.251^b^		2.864	0.239^b^
Exist	82	8.85 ± 1.84			6.21 ± 1.95			4.28 ± 1.35		
Do not exist	501	8.90 ± 1.67			5.92 ± 1.85			4.37 ± 1.37		
Uncertain	63	8.49 ± 1.96			5.86 ± 2.21			3.98 ± 1.70		
**Pandemic cognition score**	646		0.216^**^	<0.001^**^		0.271^**^	<0.001^**^		0.212^**^	<0.001^**^
**Pandemic cognition grouping**			−4.095	<0.001^a^^**^		−5.558	<0.001^a^^**^		−4.207	<0.001^a^^**^
Fine	493	9.02 ± 1.55			6.17 ± 1.85		4.45 ± 1.35			
Poor	153	8.32 ± 2.11			5.22 ± 1.86			3.89 ± 1.49		

a *Outcomes of Mann-Whitney U-test*,

b *Outcomes of Kruskal-Wallis H-test*.

** *P < 0.01*,

* *P < 0.05*.

** Indicates the Spearman's correlation for pandemic cognition is significant at the level of 0.001.

Scores for purposive rational actions were significantly affected by the major, birthplace, pandemic cognition score, and pandemic cognition grouping of respondents (*P* < 0.05). The value rational actions scores displayed significant differences in the political orientation, pandemic cognition score, and pandemic cognition grouping (*P* < 0.001). For respondents from different schools and majors, political orientations, pandemic cognition scores, and pandemic cognition grouping, the differences in affective actions scores were also statistically significant (*P* < 0.05).

### Multiple Regression Analysis of Social Behavior Scores Among Students From Chinese Medical Colleges

Except for the pandemic cognition score, the independent variables in the model were all categorical variables. After using dummy variables to assign values, the variables whose social behavior scores in each dimension were shown to be statistically significant in the single factor analysis were included in the model. The total scores of each dimension of social behaviors were taken as the dependent variables in the multiple linear regression model. According to Table 5, compared with urban areas, college students with rural birthplaces had lower scores on average for purposive rational actions (*P* < 0.05). The purposive rational actions scores of college students with better COVID-19 knowledge scores were 20.5% higher than those of college students with lower scores (*P* < 0.001) (see Table 5).

**Table 5:** The multiple linear regression results on the purposive rational actions scores of college students from Chinese medical colleges (*n* = 646).

	**Reference**	**Unstandardized coefficient**		**Standardized coefficient**	***t***	***P***	**Tolerance**	**VIF**
		**B**	**Standard error**	**β**				
(Constant)		5.196	1.043		4.982	<0.001^**^		
**Major**								
Near-peer medical students	Medical students	0.127	0.126	0.039	1.007	0.314	0.995	1.005
**Birthplace**								
Rural area	Town	−0.297	0.126	−0.091	−2.352	0.019^*^	0.990	1.010
Pandemic cognition score		0.121	0.032	0.205	3.745	<0.001^**^	0.498	2.009
Pandemic cognition Group								
Poor cognition	Good cognition	0.147	0.253	0.032	0.580	0.562	0.496	2.016

** *P < 0.01*,

* *P < 0.05*.

The results of the value rational actions model showed that medical college students with a political orientation of “probationary party member or party member” and higher scores on pandemic cognition had higher value rational actions scores (see Table 6).

**Table 6:** The multiple linear regression results on the value rational actions scores of college students from Chinese medical colleges (*n* = 646).

	**Reference**	**Unstandardized coefficient**		**Standardized Coefficient**	***t***	***P***	**Tolerance**	**VIF**
		**B**	**Standard error**	**β**				
(Constant)		1.246	1.133		1.100	0.272		
**Political orientation**								
Communist youth league member	Mass	−0.013	0.287	−0.003	−0.045	0.964	0.304	3.287
Active applicants of party membership	Mass	0.499	0.336	0.090	1.486	0.138	0.381	2.622
Probationary party member/party member	Mass	1.132	0.376	0.158	3.007	0.003^*^	0.505	1.981
Pandemic cognition score		0.150	0.035	0.251	4.333	<0.001^**^	0.417	2.399
**Pandemic cognition group**								
Poor cognition	Good cognition	−0.081	0.257	−0.018	−0.315	0.753	0.419	2.388

** *P < 0.01*,

* *P < 0.05*.

From Table 7, it can be seen that, compared with students from Sun Yet-sen University School of Medicine, students from Guangzhou University of Chinese Medicine showed 9.5% higher affective actions scores during the COVID-19 pandemic (*P* < 0.05), and students from Guangdong Medical University had lower affective actions scores by 7.8% (*P* < 0.05). Compared with medical students, near-peer medical students had lower affective actions scores by 24.9% (*P* < 0.001), indicating that pandemic cognition scores were positively related to affective actions scores.

**Table 7:** The multiple linear regression results on affective actions scores of college students from Chinese medical colleges (*n* = 646).

	**Reference**	**Unstandardized coefficient**		**Standardized coefficient**	***t***	***P***	**Tolerance**	**VIF**
		**B**	**Standard error**	**β**				
(Constant)		1.330	0.497		2.677	0.008^*^		
**School**								
Guangzhou University of Chinese Medicine	Sun Yat-sen University School of Medicine	0.361	0.142	0.095	2.534	0.012^*^	0.973	1.027
Guangdong Medical University	Sun Yat-sen University School of Medicine	−0.449	0.223	−0.078	2.017	0.044^*^	0.903	1.107
**Major**								
Near-peer medical students	Medical students	−0.701	0.109	−0.249	6.438	<0.001^**^	0.910	1.099
Pandemic cognition score		0.112	0.016	0.259	7.028	<0.001^**^	0.996	1.004

** *P < 0.01*,

* *P < 0.05*.

## Discussion

Since the outbreak of COVID-19, the virus has spread to the whole world in a short time period, becoming a major threat to public health. Medical college students are an important reserve force for pandemic prevention and control. So, it is necessary to evaluate their cognition and describe their social behaviors.

In this study, the purposive rational actions of medical college students were generally well-behaved. In detail, the adoption rate of protective behaviors out-of-home, hygiene habits, and living habits all exceeded 80%, which revealed that the vast majority of respondents had rational compliance and development with their own protection. In sharp contrast to this was the value rational actions. In the volunteer activities there was only 7.89% of the respondents participated, and the information learning and dissemination was just 31.11%. This low participation rate may be related to the complexity and sudden outbreak of the COVID-19, and professional medical staff were the first of the rescuers and participants. Medical students, as a reserve force, have not participated in the first wave of prevention and treatment during the investigation period. The epidemic has triggered a deep thinking and discussion about the importance of major in medicine in China, so in affective actions we found that the medical student group had shown higher enthusiasm than near-peer medical students in terms of professional identity and career planning actions. A study by Jan Domaradzki et al. (17) was conducted among 417 healthcare students via online questionnaire, and the result showed that future healthcare professionals expressed a strong interest in active participation during the COVID-19 outbreak.

Our subjects showed good cognition of COVID-19, and also reflected the positive promotion effect on social behaviors. The average score of subjects regarding the cognition related to COVID-19 was 30.55 ± 3.17. 493 participants had a good level of cognition, accounting for 76.32%. This suggested that students from medical colleges had a high level of cognition related to COVID-19, consistent with the results of a study conducted by Ashraf I. Khasawneh et al. (18). But there was no significant differences of most cognition items on COVID-19 knowledge betweem the medical and near-peer medical students, which was also similar with the findings of Zhiyan Gao et al. (19). And according to the study of Yuyi Zhang et al. (20), medical students' cognition of COVID-19 was better than non-medical students. These can possibly be explained by the fact that the students in medical colleges have learned more professional medical knowledges. A study by Mansour Alsoghair et al. (13) showed that academic level was the only significant factor related to the level of COVID-19 knowledge.

Meanwhile, cognition of COVID-19 pandemic was an important positive factor that significantly affected social behavior in different dimensions among medical college students. The scores for the purposive rational actions, value rational actions, and affective actions of college students with higher cognition scores were also higher than those with lower cognition scores (*P* < 0.001). This result was consistent with the research conducted by Youkun Hu et al. (21) during the SARS epidemic and Mansour Alsoghair et al. (13) during the COVID-19 pandemic. As COVID-19 may coexist with humans for a long term, the quality of relevant medical education was very important (22). To sum up, we suggest that college administrators should offer the knowledge of infectious disease prevention and control as a general course. This can improve students' awareness and promote their response, cooperation and empathy during public health emergencies.

The regression results showed that during the COVID-19 pandemic, scores for purposive rational actions of college students with rural backgrounds were lower. This was specifically manifested in the following aspects: personal hygiene habits, living habits, protective behaviors at home, and protective behaviors out-of-home. The main reasons may be: (1) The pandemic broke out during college vacation, which meant that native families had a greater influence on behavior guidance. One study showed that family support had a significant impact on individual health behaviors (23), while others found that differences in medical care between urban and rural areas, and the relative backwardness of the education level in rural areas resulted in a negative impact (24). (2) Compared to large cities, the availability of information and health resources in rural areas was relatively low. In China, the outbreak of COVID-19 mainly concentrated in large cities with higher population density. So, government allocation of health resources gave priority to these high-risk places.

The results in Table 6 revealed that medical college students who were probationary party members or party members scored higher in value rational actions. This was similar with the findings by the Chinese scholars Bian Fei and Liu Zhiwen (25). They found that party members were more proactive in relevant volunteer activities. And Jan Domaradzki et al. (17) also found that there was a significant positive relationship between students' religiosity and their eagerness to commitment for the sake of the community rather than for personal or egoistic motives in Poland. We suggest that colleges could establish more platforms for students to share and communicate with each other. It was also found that scores of medical students in affective actions were significantly higher than those of near-peer medical students. That may be related to their receipt of more medical education. Accordingly, in addition to imparting professional knowledge, medical colleges should also pass on and strengthen the professional ethics of medical students to save lives and help the wounded.

However, there are several limitations in this research. First, the survey was only conducted within Guangdong Province of China, and the sample size was not large enough. Second, the study was conducted during the pandemic and may lack vertical contrast. A further survey will be conducted to compare the cognition and social behaviors of medical college students after the pandemic. Third, the Chinese government implemented strict measures due to the raging pandemic, so the questionnaires were only submitted by participants through an online platform. Thus, the results may be biased. In future studies, more trained professionals could assist respondents to fill out the questionnaire one-by-one. Finally, due to the inherent nature of the cross-sectional design, it was difficult to establish causal relationships between variables.

## Conclusions

The study found that medical college students in China had a good level of cognition about COVID-19 knowledge. In late 2019 and early 2020, the outbreak of COVID-19 had attracted the attention of the whole world. In a short time, the Chinese government had alleviated fear of the epidemic and improved public protection level through timely and transparent information disclosure. A good cognition level of COVID-19 among medical college students had a positive impact on their social behaviors in all dimensions. Correct awareness of the epidemic could enable students to adopt more targeted and effective protective behaviors. Medical colleges should offer relevant professional courses for students to learn how to prevent and control infectious diseases. And the government should formulate targeted publicity programs to update the knowledge about the pandemic through multiple channels such as Twitter, official government websites and other mass medias. Meanwhile, because of the honor and responsibility for the organization, probationary party members and party members showed more obvious value rational actions in public health emergencies. Therefore, medical colleges should pass on and strengthen the professional ethics of medical students to save lives and help the wounded. It is also an intrinsic drive to promote their cognitive level and social engagement.

## Data Availability Statement

The original contributions presented in the study are included in the article/supplementary material, further inquiries can be directed to the corresponding author/s.

## Ethics Statement

The studies involving human participants were reviewed and approved by School of Medical Business, Guangdong Pharmaceutical University. The patients/participants provided their written informed consent to participate in this study. Written informed consent was obtained from the individual(s) for the publication of any potentially identifiable images or data included in this article.

## Author Contributions

QZ, XL, and ML are responsible for conceptualization and methodology. XL, ML, and XZ are responsible for investigation and formal analysis. XL, ML, XZ, and LY are responsible for original draft preparation. LY is responsible for editing and submission. QZ is responsible for supervision. All authors have read and agreed to the published version of the manuscript.

## Conflict of Interest

The authors declare that the research was conducted in the absence of any commercial or financial relationships that could be construed as a potential conflict of interest.

## Publisher's Note

All claims expressed in this article are solely those of the authors and do not necessarily represent those of their affiliated organizations, or those of the publisher, the editors and the reviewers. Any product that may be evaluated in this article, or claim that may be made by its manufacturer, is not guaranteed or endorsed by the publisher.
